# Supplementary material for: Nitrate Addition Increases the Activity of Microbial Nitrogen Removal in Freshwater Sediment
Source: Microorganisms. 2022 Jul 15;10(7):1429. doi: 10.3390/microorganisms10071429 (PMC9317351; doi:10.3390/microorganisms10071429)
Supplement: Supplementary file 1 [file microorganisms-10-01429-s001.zip › microorganisms-1795548-supplementary.pdf]

Supplementary Materials

Min Cai [1], Yiguo Hong [1], Jiapeng Wu [1], Selina Sterup Moore [2], Teofilo Vamerali [2], Fei Ye [1,*] and Yu Wang [1,*]

[1] Institute of Environmental Research at Greater Bay Area, Key Laboratory for Water Quality and Conservation of the Pearl River Delta, Ministry of Education, Guangzhou University, Guangzhou 510006, China; 15883918123@163.com (M.C.); yghong@gzhu.edu.cn (Y.H.); wujiapeng@gzhu.edu.cn (J.W.)

[2] Department of Agronomy, Food, Natural Resources, Animals and Environment (DAFNAE), University of Padova, 35122 Padova, Italy; selina.moore@unipd.it (S.S.M.); teofilo.vamerali@unipd.it (T.V.)

* Correspondence: yefei09007@gzhu.edu.cn (F.Y.); wangyu@gzhu.edu.cn (Y.W.)

Additional Supporting Information

Table S1 The primer sets and amplification conditions used in this study.

Figure S1 Potential rates of (a) denitrification and (b) N_2O production between three ponds under varying concentrations of NO_3^- addition.

Figure S2 Potential rates of denitrification between three ponds under varying concentrations of glucose addition.

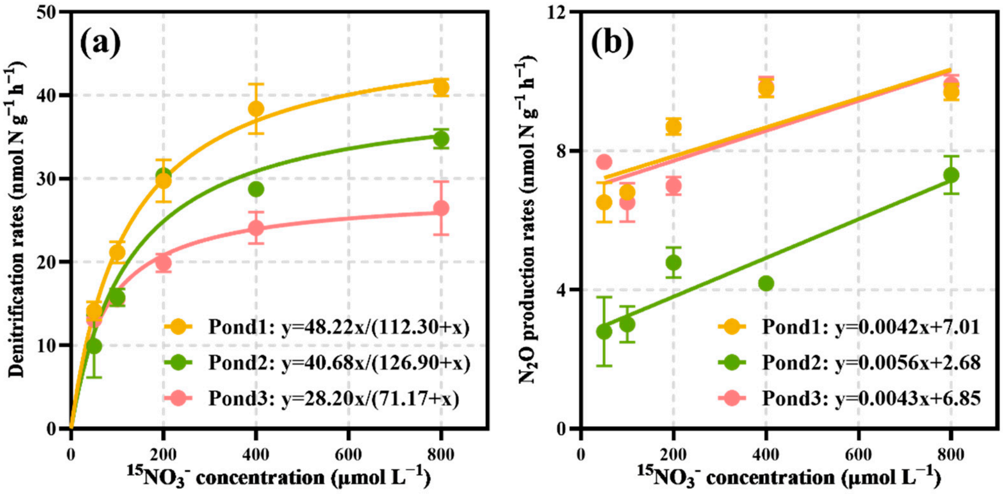

Figure S1. Potential rates of (**a**) denitrification and (**b**) N_2O production between three ponds under varying concentrations of NO_3^- addition.

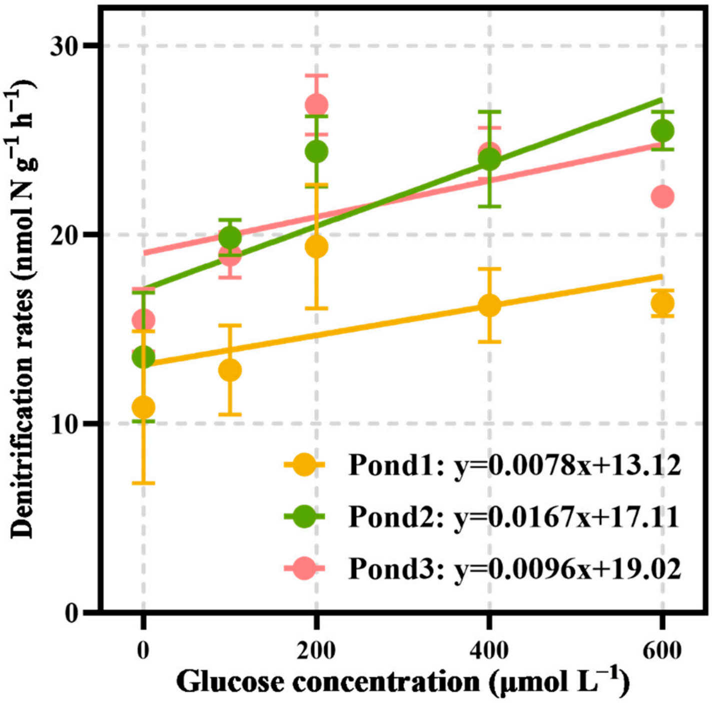

Figure S2. Potential rates of denitrification between three ponds under varying concentrations of glucose addition.

Table S1. The primer sets and amplification conditions used in this study.

Target Gene	Primer	Primer Sequence (5′-3′)	Size (bp)	qPCR Program	References
Anammox 16S rRNA	438F	GTCRGGAGTTADGAAATG	246	95°C for 3 min, 40 × (95°C for 15 s, 56°C for 30 s, 72°C for 30 s)	[1]
	684R	ACCAGAAGTTCCACTCTC			
nirS	cd3aF	GTSAACGTSAAGGARACSGG	426	95°C for 10 min, 40 × (95°C for 15 s, 56°C for 45 s, 72°C for 45 s)	[2]
	R3cd	GASTTCGGRTGSGTCTTGA			
nirK	Flacu	ATCATGGTSCTGCCGCG	473	95°C for 2 min, 36 × (95°C for 30 s, 56°C for 45 s, 72°C for 45 s)	[3]
	R3cu	TTGGTGTTRGACTAGCTCCG			
nosZ I	*nosZ*2F	CGCRACGGCAASAAGGTSMSSGT	267	95°C for 5 min, 40 × (95°C for 30 s, 68°C for 1 min, 72°C for 1 min)	[4]
	*nosZ*2R	CAKRTGCAKSGCRTGGCAGAA			
nosZ II	*nosZ* F	CTIGGICCIYTKCAYAC	746	95°C for 2 min, 40 × (95°C for 15 s, 60°C for 1 min, 72°C for 1 min)	[5]
	nosZ R	GCIGARCARAAITCBGTRC			

References

1. Han, P.; Gu, J.-D. More refined diversity of anammox bacteria recovered and distribution in different ecosystems. *Appl. Microbiol. Biotechnol.* **2013**, *97*, 3653–3663.
2. Yergeau, E.; Kang, S.; He, Z.; Zhou, J.; Kowalchuk, G.A. Functional microarray analysis of nitrogen and carbon cycling genes across an Antarctic latitudinal transect. *ISME J.* **2007**, *1*, 163–179.
3. Hallin, S.; Lindgren, P.E. PCR detection of genes encoding nitrite reductase in denitrifying bacteria. *Appl. Environ. Microbiol.* **1999**, *65*, 1652–1657.
4. Henry, S.; Bru, D.; Stres, B.; Hallet, S.; Philippot, L. Quantitative detection of the *nosZ* gene, encoding nitrous oxide reductase, and comparison of the abundances of 16S rRNA, *narG*, *nirK*, and *nosZ* genes in soils. *Appl. Environ. Microbiol.* **2006**, *72*, 5181–5189.
5. Jones, C.M.; Graf, D.R.H.; Bru, D.; Philippot, L.; Hallin, S. The unaccounted yet abundant nitrous oxide-reducing microbial community: a potential nitrous oxide sink. *ISME J.* **2013**, *7*, 417–426.
